# Supplementary material for: Functional Characterization of Aluminum (Al)-Responsive Membrane-Bound NAC Transcription Factors in Soybean Roots
Source: Int J Mol Sci. 2021 Nov 27;22(23):12854. doi: 10.3390/ijms222312854 (PMC8657865; doi:10.3390/ijms222312854)
Supplement: Supplementary file 1 [file ijms-22-12854-s001.zip › ijms-1449244-supplementary.pdf]

Table S1. Gene loci, exon and intron number, length of ORF, and protein size were extracted from the Phytozome website (http://www.phytozome.net).

Gene name	Gene Loci	Gene Bank accession no.	Exon/ Intron number	Length of ORF (bp)	Number of amino acids	Molecular weight (KDa)	Theoretical pI
GmNTL1	Glyma.02G 222300	NP_00127628 7.1	6/5	1767	589	65.6	4.88
GmNTL2	Glyma.02G 240500	XP_00351836 2.1	6/5	1929	643	73.3	5.26
GmNTL3	Glyma.04G 226700	NP_00127621 1.2	6/5	1809	603	67.6	5.25
GmNTL4	Glyma.06G 138100	XP_00352677 1.1	6/5	1794	598	67.1	5.72
GmNTL5	Glyma.07G 047900	XP_00352986 5.1	6/5	1491	497	56.7	5.77
GmNTL6	Glyma.10G 197600.1	XP_00355710 0.3	7/6	1344	448	49.7	4.50
GmNTL7	Glyma.10G 219600	NP_00127437 5.1	5/4	1680	560	62. 8	4.70
GmNTL8	Glyma.11G 212400.1	XP_01461969 0.1	6/5	2016	672	75. 9	5.40
GmNTL9	Glyma.12G 186900.1	KAG4981079	8/7	1479	493	56.1	9.14
GmNTL10	Glyma.13G 314600	KAH1104308	4/3	1113	371	42.0	7.10
GmNTL11	Glyma.14G 189300	XP_00659641 2.1	6/5	1770	590	65.8	4.88
GmNTL12	Glyma.14G 210000	XP_00354435 2.1	6/5	1932	644	73.4	5.08
GmNTL13	Glyma.18G 043900	XP_00355288 8.2	6/5	2034	678	76.5	5.52
GmNTL14	Glyma.20G 172100	XP_00355618 0.1	5/4	1647	549	61.7	4.75
GmNTL15	Glyma.20G 192300	XP_00355630 2.1	6/5	1812	604	66.9	4.82

Table S2. Primers used for vector constructions and qRT-PCR verification.

Names	Gene Loci	Forward Primers	Reverse Primers
GmNTL1	Glyma.02G222300	GTCGTCGAGTGTTACCCACA	CGTAGCTCGGTTCAACCTGT
GmNTL2	Glyma.02G240500	TTCAGATTCCACCCTACAGACG	GATTTCCCTGGCAAGTCCC
GmNTL3	Glyma.04G226700	ATGGGTATTGGAAGGCGACG	TGTGGAGTAATAGGCAGCCAC
GmNTL4	Glyma.06G138100	GCCGTTAGGGTTCCGTTTC	GCTCCTTCAAAGTGGGACGATA
GmNTL5	Glyma.07G047900	GGGAATTGAGGCACCAACCT	ATCTTCCAGCCCAATGCCAG
GmNTL6	Glyma.10G197600.1	GATTGCTCTTGGACTTCA	AGTAATGTCTGTTATGCTGAT
GmNTL7	Glyma.10G219600	CCCAAGTGCATCAGAAGGCT	GCAGTGTCTTTACCTGCTGC
GmNTL8	Glyma.11G212400.1	CAATATGTGCGGAGGAAG	GTGATGATGAGTAGTTGGATT
GmNTL9	Glyma.12G186900.1	TTCGAGGGAGGCTCAGATGT	TGGCTGGTAAATCCCAAGGC
GmNTL10	Glyma.13G314600	TTCTGCCCTACCGATGAGGA	TTTCGCTCTTTGCCAGTTGC
GmNTL11	Glyma.14G189300	GCCGTTAGGGTTCCGTTTC	AAGGCTCCCATTTGCAGACA
GmNTL12	Glyma.14G210000	CATGGATATTTGCTCACCCTGC	AGGCTTGTGCCATTTTCAGC
GmNTL13	Glyma.18G043900	ATCAATGGTGGGACAAGGGAT	TCAGCTCGGAAATGCCAACT
GmNTL14	Glyma.20G172100	CCCGGAGAGCACTTTATTTTGG	ACCGGTTCTCGATCCATAGTTG
GmNTL15	Glyma.20G192300	AGAAGGAGTGGGATGTTGAGG	ATGTCAAGGTCGTTGGTTTCA
GmEF1-α	Glyma.17G186600	TGCAAAGGAGGCTGCTAACT	CAGCATCACCGTTCTTCAAA
AtEF-α	At5G60390	GTCGATTCTGGAAAGTCGACC	AATGTCAATGGTGATACCACGC
GOLS4	At1g60470	GAAAACATGGAGCCACTCGC	GTGGTGTGATCTCCAGGGTC
At2g33160	At2g33160	TTGCGTTTACAAAGGCGTGG	ATCCACCGCCAGTAACAGTG
ALMT12	At4g17970	CGGCAAAGTCCTTCGAGCTA	GACAGATTCCGCAACCAACG
XTH33	At1g10550	GTGACACGCCTCACAGATCA	ACCACAACACCAGAGGCAAA
PME59	At5g51490	CCGGCCTTGGATGAAGTTCT	AAGTGAAGGGTATGCCGGTG
CSLB5	At4g15290	TTGGTGAATCTTGCCGCTCT	ACAAACATTAGCCTAGGGTCGG
PMDH2	At5g09660	AGCTCATCTCACTCCTCAGGT	AGCGAATGTCAAGTTCATGGT
--	At2g43880	TGGACGAGCCTCTGGAGTTA	GCACAAGCACATTGATAAGCGA
--	At2g43890	ACGGCTGCTTCCAACTACAA	ATTCTACTGCGGCATGGACC

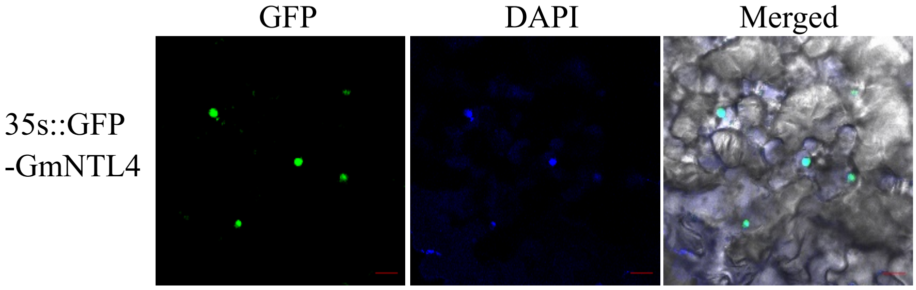

Figure S1. Subcellular localization analysis of GmNTL4 in tobacco leaves at 72 h after transformation. Green fluorescence was derived from 35S::GFP-GmNTL4, while blue fluorescence was derived from DAPI, indicating the nucleus.

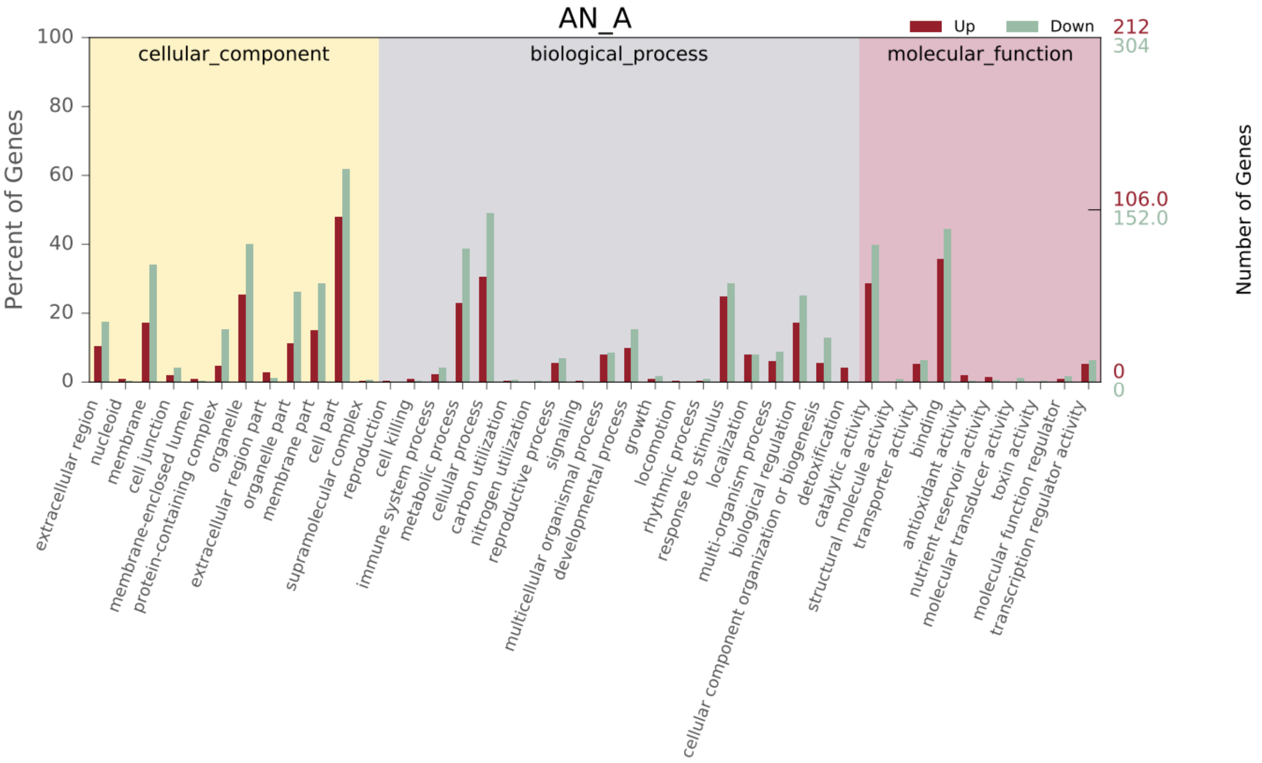

Figure S2. Gene Ontology (GO) analysis of differentially expressed genes in *GmNTL4* overexpressing *Arabidopsis*.

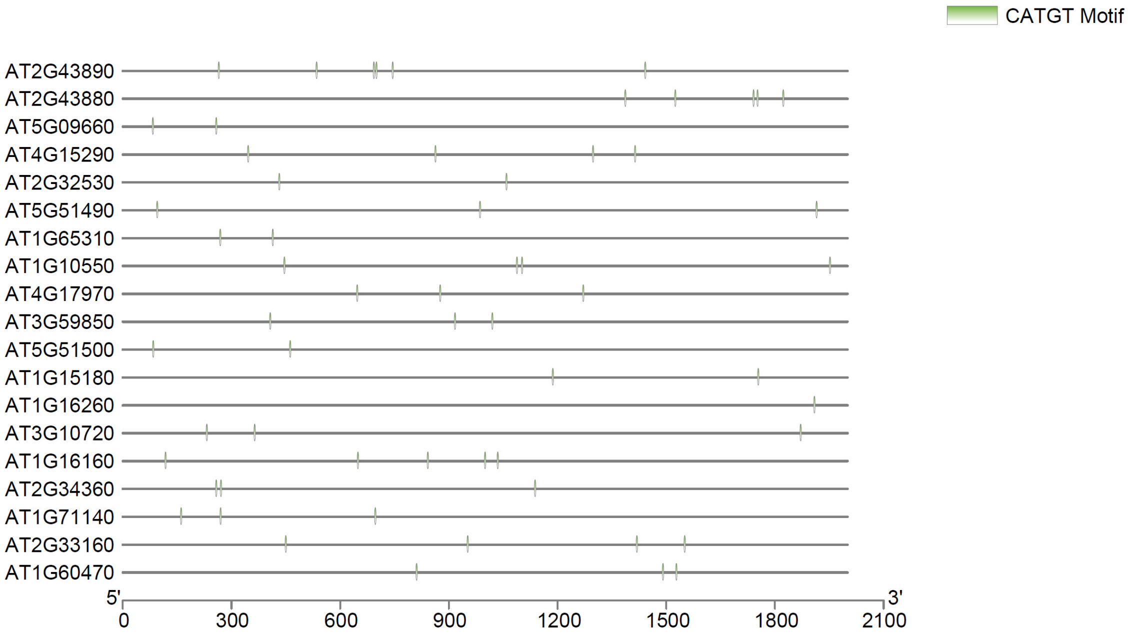

Figure S3. Putative NAC binding CATGT motifs found in the promoter regions of the selected genes regulated by GmNTL4 in Arabidopsis. The 2000 bp upstream sequence from the translation initiation site was obtained from the phytozome website (http://www.phytozome.org/index.php). The number and location of the promoter CATGT element of the selected genes were analyzed through the SOGO website (https://sogo.dna.affr.go.jp/cgi-bin/sogo.cgi). TBtools software was used to visualize the above information data.
